# Item-level measurement properties of the pediatric awareness and sensory motor assessment in children with medical complexity

**DOI:** 10.3389/fped.2025.1479298

**Published:** 2025-01-29

**Authors:** Brooke Mulrenin, Bryant A. Seamon, Carrie Cormack, Kimberly Lane Kascak, Cynthia Dodds

**Affiliations:** ^1^ Division of Occupational Therapy, College of Health Professions, Medical University of South Carolina, Charleston, SC, United States; ^2^ Division of Physical Therapy, College of Health Professions, Medical University of South Carolina, Charleston, SC, United States; ^3^ College of Nursing, Medical University of South Carolina, Charleston, SC, United States; ^4^ Office of Interprofessional Initiatives, Medical University of South Carolina, Charleston, SC, United States

**Keywords:** children with medical complexity (CMC), Pediatric Awareness and Sensory Motor Assessment (PASMA), Rasch analysis, item response theory (IRT), pediatric assessment, assessment validation

## Abstract

**Introduction:**

Children with medical complexity (CMC) are medically fragile with severe brain damage and chronic conditions, necessitating daily care. Their neurological impairments often limit participation in childhood activities, affecting quality of life. Current assessment tools fail to detect subtle abilities in CMC, hindering development of effective rehabilitation goals and interventions. The Pediatric Awareness and Sensory Motor Assessment (PASMA) was created to fill this gap, providing sensitive measurement of sensory awareness and motor response across five domains (i.e., olfactory, visual, auditory, gustatory, and tactile).

**Methods:**

In this retrospective study, a Rasch analysis was conducted on PASMA data for CMC. The PASMA was administered five times over ten weekdays to each child, reflecting its intended clinical use to gain a reliable sense of each child's awareness.

**Results:**

Analysis of data from 36 CMC revealed that the PASMA is sufficiently unidimensional, effectively measuring sensory awareness and motor response as a single construct. Its rating scale structure was validated without modifications, and the item hierarchy matched clinical expectations. High item reliability (0.97) was observed, with one item (V2 blink in response to light) slightly misfitting, but without affecting overall measures. Adequate person reliability was observed (0.81), with 15% person misfit. Person misfit did not degrade item measures or model statistics. Differential item functioning (DIF) was noted for the three easiest items on specific days. The PASMA successfully stratified participants into three distinct awareness levels (low, medium, and high awareness), without floor or ceiling effects.

**Discussion:**

The PASMA is a valid unidimensional measure of sensory awareness and motor response in CMC. Rating scale characteristics, item hierarchy, and person separation measures all support the PASMA's measurement properties within this heterogeneous sample of CMC. DIF findings support a potential reduction in the recommended number of PASMA administrations per individual. Future research will focus on establishing rater reliability and external validity. Additional efforts will support health professionals to utilize the PASMA for baseline assessments, guiding personalized interventions, and tracking progress.

**Conclusion:**

Clinical use of the PASMA could provide new opportunities to detect subtle abilities, preferences, and changes in CMC, to promote meaningful participation and improve quality of life.

## Introduction

Children with medical complexity are a medically fragile subset of children who have severe brain damage, chronic medical conditions, and persistent intensive daily care needs. Their origins of brain damage are varied and primary health conditions for these children include but are not limited to severe forms of cerebral palsy, traumatic brain injury, hypoxic encephalopathy, and genetic diseases (1, 2). Their needs commonly include complex medication regimens and dependence on medical equipment and technology including wheelchairs, ventilators, and feeding tubes.

Advances in medical technology have led to increased survival of children with medical complexity. Unfortunately, the neurological damage associated with medical complexity causes severe cognitive and motor disabilities. For this reason, children with medical complexity have intensive daily care needs and require special education programs, social services, and coordinated medical and rehabilitative care (3). Additional sequalae of medical complexity include unmet health care needs, family stress, limited participation in typical childhood occupations, and compromised quality of life (1, 4, 31).

Interprofessional health care teams including rehabilitation providers (i.e., physical therapists, occupational therapists, and speech language pathologists) can play an essential role in providing family support, and improving health, participation, and quality of life for children with medical complexity. However, the assessment tools currently used with this population do not offer adequate clinical information to guide long-term rehabilitation decision-making. For instance, imaging and electroencephalogram (EEG) are used to detect structural neurological impairment in children with brain damage, but they do not provide reliable information about functional capabilities or impairments for this population (5). Broad classifications of brain damage like the Glasgow Coma Scale (GCS) (6–8), the JFK Coma Recovery Scale-Revised (CRS-R) (9, 10), and The Rancho Levels of Cognitive Functioning Scale (LCFS) (11) provide important information in the acute phases of treatment. However, they do not have the sensitivity to identify subtle sensory awareness and motor behaviors.

Without appropriate assessment tools, interprofessional team members miss opportunities to develop appropriate goals and enhance participation and quality of life for this population. As a result, children with medical complexity commonly receive passive and custodial care instead of appropriate individualized care. Therefore, to improve care for children with medical complexity, there is an urgent need for an assessment that measures subtle abilities including nuanced signs of sensory awareness and purposeful motor behaviors. To meet this need, the Pediatric Awareness and Sensory Motor Assessment (PASMA) was developed by an interprofessional team of clinicians, therapists, and researchers who serve children with medical complexity. This is the first publication about this assessment.

The PASMA is an observation-based assessment designed to capture subtle signs of awareness of/response to sensory stimulation across multiple sensory domains, to help health professionals gain baseline knowledge of the child's awareness, guide individualized interventions, and monitor changes over time. The instrument was modeled after a similar tool for adults with severe brain damage, the Sensory Modality Assessment and Rehabilitation Technique (Adult SMART) (12). The Adult SMART is more effective at discriminating awareness and detecting higher levels of cognitive functioning in individuals with severe neuro-disability compared to other tools used with this population, including the Western Neuro Sensory Stimulation Profile (WNSSP) (13, 14), the JFK Coma Recovery Scale (15), and the Sensory Stimulation Assessment Measure (13, 16).

The purpose of the present study was to examine item-level measurement properties of the PASMA using Rasch analysis. Unlike classical test theory which evaluates psychometric properties of an instrument as a whole, Rasch analysis examines measurement properties of each individual item within an assessment (17). Rasch analysis uses probabilistic mathematical modeling to examine a tool's ability to quantify abstract constructs in a meaningful way (18), which is especially useful for assessments that are based on clinician observation or patient report rather than physiological measurement (17, 32). Rasch analysis can be conducted when items within an assessment represent a single construct and a reasonable hierarchy of item difficulty can be presumed. When analyzing a dataset, the Rasch mathematical model assigns each assessment item a “difficulty” score and each person an “ability score”, and the probability that each person will be successful on each item is calculated based on these scores (17). The model produces multiple metrics of measurement properties for each assessment item and produces an ordered version of the assessment with item difficulty set on a linear scale (17).

We hypothesized that the PASMA's measurement characteristics would align with Rasch measurement theory as evidenced by measurement unidimensionality, an appropriate rating scale system, and an item hierarchy that aligns with clinical and theoretical expectations.

## Methods

### Data source

Study methodology was informed by the RULER statement (33). The present study is a retrospective secondary analysis of clinical data collected during a prospective descriptive cohort study of children with medical complexity. Informed consent was obtained for participation in the prospective study. Deidentified data were used in the present study and IRB approval of this secondary analysis was not required (19).

Medically stable children with medical complexity between the ages of 5 and 21 years old were eligible for inclusion in this study. Children with uncontrolled life-threatening diseases were excluded.

### Instrument

The PASMA consists of 30 scored items, and additional unscored components (e.g., a baseline observation period and documentation of observed side preferences for 17 items, described below) that provide important information about the child. Before beginning the scored portion of the PASMA, the child is seated in a quiet exam room for a five-minute baseline observation period. During this period, the assessor observes and documents the child's alertness and common motor behaviors (e.g., specific voluntary and involuntary movements). After the baseline observation period, the 30 scored items are administered. These items are structured across five sensory domains: Olfactory (four items), visual (10 items), auditory (two items), gustatory (six items), and tactile (eight items). Table 1 shows all PASMA items listed by sensory domain.

**Table 1:** All pasma items listed by sensory domain.

Olfactory items
O1 mint scent
O2 cinnamon scent
O3 lemon scent
O4 preferred scent motor response^b^
Visual items
V1 pupillary light response^b^
V2 blink in response to light^b^
V3 visual threat finger^b^
V4 visual threat red pompom^b^
V5 near vision upper quadrants^a^^,^^b^
V6 near vision lower quadrants^a^^,^^b^
V7 horizontal tracking^a^
V8 vertical tracking^a^
V9 preferred quadrant vision with sound^a^
V10 visually guided reach
Auditory items
A1 40 dB low intensity sound^a^^,^^b^
A2 70 dB conversational intensity sound^a^^,^^b^
Gustatory items
G1 sweet taste
G2 salty taste
G3 sour taste
G4 cold temperature on lips
G5 seeking taste lip gloss
G6 anticipatory mouth opening
Tactile items
T1 feather touching face^b^
T2 feather touching hand^b^
T3 monofilament touching face^b^
T4 monofilament touching hand^b^
T5 pin prick on hand^b^
T6 pin prick on foot^b^
T7 ice touching face^b^
T8 ice touching hand^b^

^a^ Denotes the seven items that use a three-point rating scale (0, 1, 2). The other 23 items use a 2-point rating scale (0, 2).

^b^ Denotes items that are tested on both sides of the body. In PASMA scoring for these items, only the higher score counts (i.e., score from testing on the preferred side), but performance on both sides and observed side preferences are also recorded on the score sheet for clinical use.

For 23 PASMA items, scoring is dichotomous (i.e., 0 = No response OR the child's commonly observed motor response, meaning no response beyond baseline motor patterns; 2 = a discrete response to the sensory information presented). The other seven PASMA items use a three-point rating scale (i.e., 0 = least possible response; 1 = medium response; 2 = best response). Table 2 shows examples of administration and scoring directions for select PASMA items.

**Table 2:** Directions for administration and scoring of select PASMA items.

OLFACTORY- *O1 mint scent; O2 cinnamon scent O3 lemon scent*
Directions: Without touching the nose, place 1 of Sanford Mr. Sketch Scented Marker beneath the nostrils of the child for 30 s Remove stimulus once response occurs. Complete for 4 markers. Allow a 30 s rest between scents. Mint scent Cinnamon scent Lemon scent Scoring: 0 = No response OR commonly observed motor response 2 = Localized response: nasal flaring, increased inhalation depth, increased inhalation rate, lip smacking, teeth grinding, chewing, tongue thrust, increased drooling, increased sucking, increased swallowing, smiling, pouting, grimacing, quieting or exaggeration of movement, appears to attend
VISION - *V4 visual threat red pompom*
Directions: Pinching a small red pompom, draw a “+” within 1 inch of the right eye. Enter and exit from lateral aspect of right eye. Do not touch eyelashes or create a breeze. Scoring: 0= No response OR commonly observed motor response 2= Increased blinking, look towards or away from stimulus, turn head toward or away from stimulus
AUDITORY - *A2 70 dB conversational intensity sound*
Directions: Have the nurse, parent, or available individual stand behind the child so he/she cannot see the individual. Approximately 12 inches from right ear, ask the individual to clearly child's name in an indoor conversational voice. Scoring: 0 = No response 1 = Commonly observed motor response 2 = Turns eyes or moves head in direction of sound, quieting of common motor response, appears to attend
GUSTATORY *- G5 seeking taste lip gloss*
Directions: Apply lip-gloss to lips with a clean Q-tip. Observe for 30 s and score. Scoring: 0 = No response OR commonly observed motor response 2 = Tongue seeks stimulus on lips
TACTILE - *T3 monofilament touching face; T4 monofilament touching hand*
Directions: Apply 4.31 (protective sensation) filament to locations. Apply pressure to facilitate bending of the filament and maintain bend for 1.5 s. Monitor response for 30 s. If no response, apply 3 consecutive repetitions of filament. Monitor response for 30 s. Right face Left face Right hand Left hand Scoring: 0 = No response OR commonly observed motor response 2 = Smiling, frowning, grimacing, pouting, blinking, eyes open or close, vocalizations, withdrawal or seeking of stimuli, quieting of movement, appears to attend

This table shows examples of administration and scoring directions for select PASMA items from each sensory domain.

Importantly, 17 PASMA items are administered on both the right and left side of the body (i.e., one olfactory item, six visual items, two auditory items, and eight tactile items) because a participant's responsiveness to unilateral stimuli will vary depending on their specific brain damage. Only the participant's best response for each item (i.e., response on the preferred side) is included in the PASMA score (see Table 3). Therefore, the item scores included in the Rasch analysis represent each participant's best sensory awareness and motor response (i.e., when all stimuli are presented on their preferred side). However, the participant's performance on both sides and any observed side preferences are recorded on the PASMA score sheet for use by clinicians and caregivers. Additionally, any other observed sensory preferences may be documented but are not scored (i.e., a preferred color on visual items, preferred scent on olfactory items, or preferred taste on gustatory items).

**Table 3:** Deriving a participant's PASMA scores for select items tested on both sides of body.

Item	Performance (for stimuli on each side of body)^a^	Observed side preference	PASMA score (participant's best response)
T1 feather touching face		No preference	T1 feather touching face = **2**
a. Right side	a. 0, **2**	Right preference	
b. Left side	b. **0**, 2	Left preference	
T2 feather touching hand		No preference	T2 feather touching hand = **0**
a. Right side	a. **0**, 2	Right preference	
b. Left side	b. **0**, 2	Left preference	
T3 monofilament touching face		No preference	T3 monofilament touching face = **2**
a. Right side	a. 0, **2**	Right preference	
b. Left side	b. 0, **2**	Left preference	
T4 monofilament touching hand		No preference	T4 monofilament touching hand = **2**
a. Right side	a. 0, **2**	Right preference	
b. Left side	b. **0**, 2	Left preference	

Of the 30 PASMA items, 17 items are administered on both the right side and left side of the body. This table presents one participant's performance, side preference, and scores on items T1-T4 (in bold) to illustrate (1) how observed side preferences were documented for clinical use and (2) how PASMA scores used for this analysis were derived. For example, tactile items T1a and T1b each receive their own performance rating: 2 for the right side and 0 for the left side, respectively. The PASMA score for T1 is a 2, representing the participants best performance, and the right preference observed for this item is documented on the assessment score sheet.

^a^ For items shown in Table 3, 0 = no response OR the child's commonly observed motor response; 2 = a discrete response to the sensory information presented.

Like the Adult Smart, the PASMA was designed to be administered five times per participant, on five different days within 10 calendar weekdays. Multiple administrations are recommended because conditions that elicit each child's best performance (e.g., time of day and alertness) vary. This administration schedule allows the assessor to gain a thorough understanding of an individual's sensory awareness and motor functioning within a relatively short timeframe (12).

### Tool administration

All but two participants in this sample completed testing on all five days; two completed less than five days of testing (completed 2 and 3 days) due to acute medical issues. Each participant who completed 5 days of testing was either seen in the morning for 3 days and the afternoon for 2 days, or vice versa. The time of day was recorded, but all scores from each PASMA administration were included in the Rasch analysis without controlling for time of day. This design was selected to examine the strength of item-level measurement properties irrespective of participant alertness level, to ensure a robust instrument. To examine whether dependent pairs of data impacted results, Differential Item Functioning was examined across the 5 assessment days.

### Rasch analysis

Rasch analysis of the PASMA was completed with the Rating Scale Model in Winsteps version 3.93.1 (20).

#### Dimensionality

The Rating Scale Model assumes unidimensionality of the instrument, meaning that all items on the instrument examine the same measurement construct. We hypothesized the PASMA captures a single comprehensive measurement of a child's sensory and motor awareness that consists of the five sensory domains. We tested this with a principal components analysis of residuals (21). The following criteria were used to determine whether unidimensionality was sufficient for measurement: (1) at least 50% variance in the data is explained by the Rasch dimension, (2) the first contrast in residuals explains <∼4% of data variance, and (3) the size (Eigenvalue) of the first contrast in the standardized residuals is ≤2 (22). If criteria were not met (i.e., suggesting multiple dimensions exist), we examined item clusters for additional dimensions and determined whether clusters made sense clinically and theoretically.

We also compared disattenuated correlations of additional dimensions with the Rasch dimension, to determine whether multiple dimensionality affected person measurement. Disattenuated correlations >0.82 were considered to represent dependent item clusters which were sufficiently unidimensional and not degrading measurement (22).

Additionally, we tested the Rasch model assumption that items on the instrument are locally independent. Items were considered locally independent if correlations between standardized residuals of items did not exceed 0.7 (33).

#### Rating scale structure

Linacre's three rating scale criteria were used to determine the appropriateness of rating scale categories: (1) Each rating scale category has a minimum of 10 observations; (2) The average measures of rating scale categories advance monotonically; and (3) Outfit mean square values are less than 2.0 (23).

#### Item fit statistics and person fit statistics

Item and person fit statistics were compared to previously established criteria to identify possible misfit (24). Items and individuals were classified as misfitting the Rasch model if outfit statistics had mean square standardized residuals ≥1.4 and standardized *z*-scores ≥2. Misfitting items were removed to examine the effect on remaining item fit and to determine whether their removal affected person ability measures. Items with misfit were retained if removing them caused additional items to misfit and/or if removing the item did not have a meaningful effect on person ability measures.

If >10% of persons in the sample were found to misfit the model's expectations for item response patterns, the effect of this misfit was explored by removing persons with the most unexpected patterns from the dataset to determine whether this affected item difficulty measures. Misfitting persons were retained if removal did not influence item difficulty measures.

#### Item difficulty hierarchy

The Rasch model produces item difficulty measures and person ability measures on the same interval scale using logits. Lower measures are assigned to easier items and persons with less ability. Higher measures are assigned to harder items and persons with more ability. Thus, conducting a Rasch analysis produces a hierarchy of item difficulty, based on the measure estimates of item difficulty. We examined this hierarchy to determine whether it was consistent with clinical and theoretical expectations (25).

#### Floor and ceiling effects

We examined the distribution of person ability in our sample and assessed for floor effects and ceiling effects, defined as >15% of individuals in the sample having the minimum or maximum possible score, respectively (26).

#### Differential item function

Tests of differential item function (DIF) were used to test whether the test administration day had an effect on item difficulty measures. Items were considered to have DIF if item difficulty measures were different by a magnitude of 0.5 logits and had an associated t-statistic that was >2 or <−2 (27).

#### Separation index

Person separation index was used to evaluate the assessment's ability to separate people into statistically distinct strata. The formula below was used to calculate the number of strata in our sample (28):

Strata=[4×(personseparationindex)+1]/3

## Results

### Participant characteristics

Thirty-six children with medical complexity participated in the original data collection study and all PASMA data collected from these participants was included in the present analysis. Participants were between 5 and 21 years old with a mean age of 11 years. The sample included 11 females (31%) and 25 males (69%), and multiple races and ethnicities were represented (see Table 4). All participants (100%) had severe brain damage, but the origins of brain damage varied; primary health conditions included cerebral palsy, traumatic brain injury, hypoxic encephalopathy, and genetic diseases. At the time of testing, all children were confirmed by their physician or nurse to be medically stable, defined as having life-threatening diseases under control. All 36 participants (100%) were wheelchair users and were dependent on caregivers for mobility, communication, and daily care needs (i.e., eating, bathing, grooming, dressing, toileting,). In addition to primary diagnoses, many participants had co-occurring medical conditions (e.g., 42% had a history of seizures) and many required specific medical support for survival (e.g., 75% used a gastrostomy tube for feeding and 11% used a tracheostomy tube for breathing).

**Table 4:** Participant characteristics.

Total Sample *n* = 36 (100)			
Age (years)			
Mean	11		
Range	5–21		
Mode	10		
Sex			
Female	11 (31)		
Male	25 (69)		
Race			
American Indian	1 (3)		
Asian	1 (3)		
Native Hawaiian or Other Pacific Islander	0 (0)		
Black/African American	7 (19)		
White	19 (53)		
Unknown/not specified	8 (22)		
Ethnicity			
Hispanic/Latino	7 (19)		
Not Hispanic/Latino	29 (81)		
Co-occurring conditions and supports			
Seizures	15 (42)		
Tracheostomy	4 (11)		
Wheelchair propelled by caregiver	36 (100)		
Gastrostomy tube (G-tube)	27 (75)		
Cerebral Palsy (CP) Subgroup *n* = 20 (56)^a^	Ability levels by CP classification systems		
	III	IV	V
GMFCS Level	0 (0)	1 (5)	19 (95)
MACS Level	3 (15)	2 (10)	15 (75)
CFCS Level	0 (0)	2 (10)	18 (90)
EDACS Level	1 (5)	4 (20)	15 (75)

Continuous variables are presented as frequency count (percentage). GMFCS, gross motor classification system; MACS, manual ability classification system; CFCS, communication function classification system; EDACS, eating and drinking ability classification system. All Cerebral Palsy (CP) Classification Systems listed include five distinct levels of ability (I to V). For each classification system, Level I represents the most ability (least impairment) and Level V represents the least ability (most impairment). Participants with CP in this sample had ability levels between III and V on all CP classification system scales.

^a^ Primary health conditions for the 16 participants without CP (44%) included traumatic brain injury, hypoxic encephalopathy, and genetic diseases.

#### Dimensionality

The principal components analysis showed that the Rasch dimension explained 46.0% of the variance in our data, which did not meet the criterion of >50%. The first contrast in residuals explained 6.2% of the variance in our data, which did not meet the criterion of <4%. The corresponding eigenvalue was 3.4, which did not meet the criterion of <2.0.

Because criteria for unidimensionality were not met, we examined additional clusters to determine whether there was a clinical/theoretical explanation. We found that the secondary dimension consisted of visual items and was therefore explained by these items representing a specific sensory system. We examined the disattenuated correlations between the primary dimension (i.e., the Rasch dimension) and secondary dimensions, which was 1.0 (meeting the guideline of >0.82). This indicates that these two clusters of items are dependent and therefore, can be treated as representative of the same latent construct without degrading measurement quality (29). Therefore, the PASMA is sufficiently unidimensional for measuring sensory awareness/motor response, and all items can be considered a single construct.

Correlations between standardized residuals of items did not exceed 0.7 for any item pairs, meeting the assumption of local independence.

#### Rating scale structure

Rating scale structures for PASMA items met the three essential criteria. (1) Each rating scale category had >10 observations. (2) The rating scale categories advanced monotonically, and (3) Outfit mean square values are <than 2.0. The rating scale structure for the PASMA, observed averages for each score, and associated infit and outfit mean square values are shown in Table 5.

**Table 5:** Rating scale structure of the PASMA.

Score	Frequency count (%)	Observed average	Infit mean-square	Outfit mean-square
0	198 (17)	−0.70	1.00	0.99
1	427 (36)	0.35	0.86	0.75
2	570 (48)	1.85	0.90	0.91
Missing	44 (4)			

This table shows the rating scale structure for PASMA items and demonstrates that the measures advance monotonically, represented by increasing observed average values associated with higher scores. Associated infit and outfit mean square values are shown and these values fit the Rating Scale Model.

#### Item fit statistics and person fit statistics

**Figure 1:**
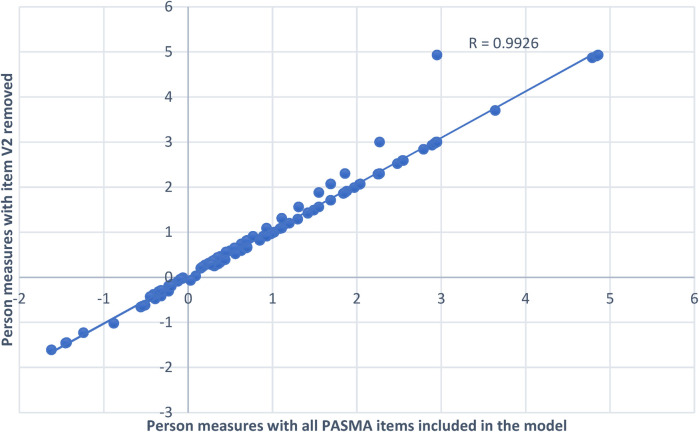
Comparing person ability measures with vs. without misfitting item (V2 blink in response to light) in the model. The *x*-axis of this figure displays person ability measure estimates with all PASMA items included in the model. The *y*-axis displays person ability measures estimates when the one misfitting item (V2 blink in response to light) was removed from the model. In spite of a few person measure outliers that occurred when item V2 was removed, person ability measures for both scenarios (with and without item V2) are very highly correlated (R = 0.9926). The line of best fit (the upward slanting dotted line) for both datasets is very close to person measure estimates with all items included in the model. Therefore, all items were left in the final analysis model.

Item fit statistics are presented in Table 6. One of the 30 PASMA items (V2 blink in response to light) misfit the Rating Scale Model, with outfit mean square values and standardized *z*-scores above our pre-specified thresholds (≥1.4 and ≥2.0, respectively). Removing this item caused another item to misfit (G5 seeking taste lip gloss). Figure 1 shows results from the comparison of person ability measures with vs. without the misfitting item. The person ability measures from these scenarios were very highly correlated (R = 0.9926), indicating that removing this item does not cause a significant change in person ability measures. Therefore, we determined that keeping this item did not degrade PASMA measurement properties.

**Table 6:** PASMA items in measure order with item Fit statistics.

Item	Measure	Model standard error	Infit		Outfit	
			Mean square	*z*-score	Mean square	*z*-score
V10 visually guided reach	1.97	0.12	1.21	1.6	1.42	1.0
V8 vertical tracking	1.83	0.13	0.70	−3.4	0.72	−2.9
V7 horizontal tracking	1.54	0.13	0.73	−3.0	0.75	−2.7
G6 anticipatory mouth opening	1.16	0.11	0.92	−0.7	0.67	−1.1
V3 visual threat finger	0.98	0.11	1.11	0.9	1.00	0.1
V4 visual threat red pompom	0.64	0.11	1.25	2.1	1.05	0.3
V6 near vision lower quadrants	0.44	0.13	0.92	−0.8	0.81	−1.7
V2 blink in response to light^a^	0.39	0.11	1.41	3.3	2.57^b^	2.3^b^
T4 monofilament touching hand	0.29	0.11	0.88	−1.1	0.63	−0.6
T2 feather touching hand	0.25	0.11	0.97	−0.2	0.85	−0.1
T5 pin prick on hand	0.14	0.12	1.19	1.5	1.17	0.5
A1 40 dB low intensity sound	0.07	0.14	1.11	1.1	1.25	1.9
O4 preferred scent motor response	0.04	0.12	0.87	−1.1	0.77	−0.1
V9 preferred quadrant vision with sound	0.03	0.14	1.24	2.1	1.01	0.1
T8 ice touching hand	0.02	0.12	0.91	−0.7	0.60	−0.4
T3 monofilament touching face	−0.01	0.12	0.88	−0.9	0.49	−0.6
V5 near vision upper quadrants	−0.05	0.14	0.78	−2.2	0.68	−2.6
G5 seeking taste lip gloss	−0.13	0.13	1.34	2.4	1.48	0.8
T1 feather touching face	−0.19	0.13	0.82	−1.4	0.49	−0.5
T6 pin prick on foot	−0.36	0.14	1.20	1.2	0.97	0.3
V1 pupillary light response	−0.44	0.14	1.26	1.5	1.04	0.4
T7 ice touching face	−0.48	0.14	0.85	−0.9	0.47	−0.3
O2 cinnamon scent	−0.51	0.14	0.95	−0.2	0.54	−0.2
G1 sweet taste	−0.62	0.16	0.97	−0.1	0.91	0.4
O3 lemon scent	−0.73	0.16	1.00	0.0	0.54	−0.1
A2 70 dB conversational intensity sound	−1.00	0.17	1.14	1.1	0.95	−0.2
G4 cold temperature on lips	−1.00	0.19	1.00	0.1	0.45	−0.2
G2 salty taste	−1.22	0.23	0.95	0.0	0.27	−0.3
G3 sour taste	−1.52	0.28	1.33	0.8	1.36	0.7
O1 mint scent	−1.54	0.25	1.03	0.2	1.02	0.4

This table presents fit statistics for all PASMA items. Items are presented in measure order, with the most difficult items at the top and the easiest items at the bottom of the table. The one item that misfit the Rating Scale Model is noted below (V2 blink in response to light) with outfit values that caused misfit.

^a^ Item misfit the Rating scale model.

^b^ Outfit mean square and *z*-score values that caused item misfit (≥1.4 and ≥2.0, respectively).

**Figure 2:**
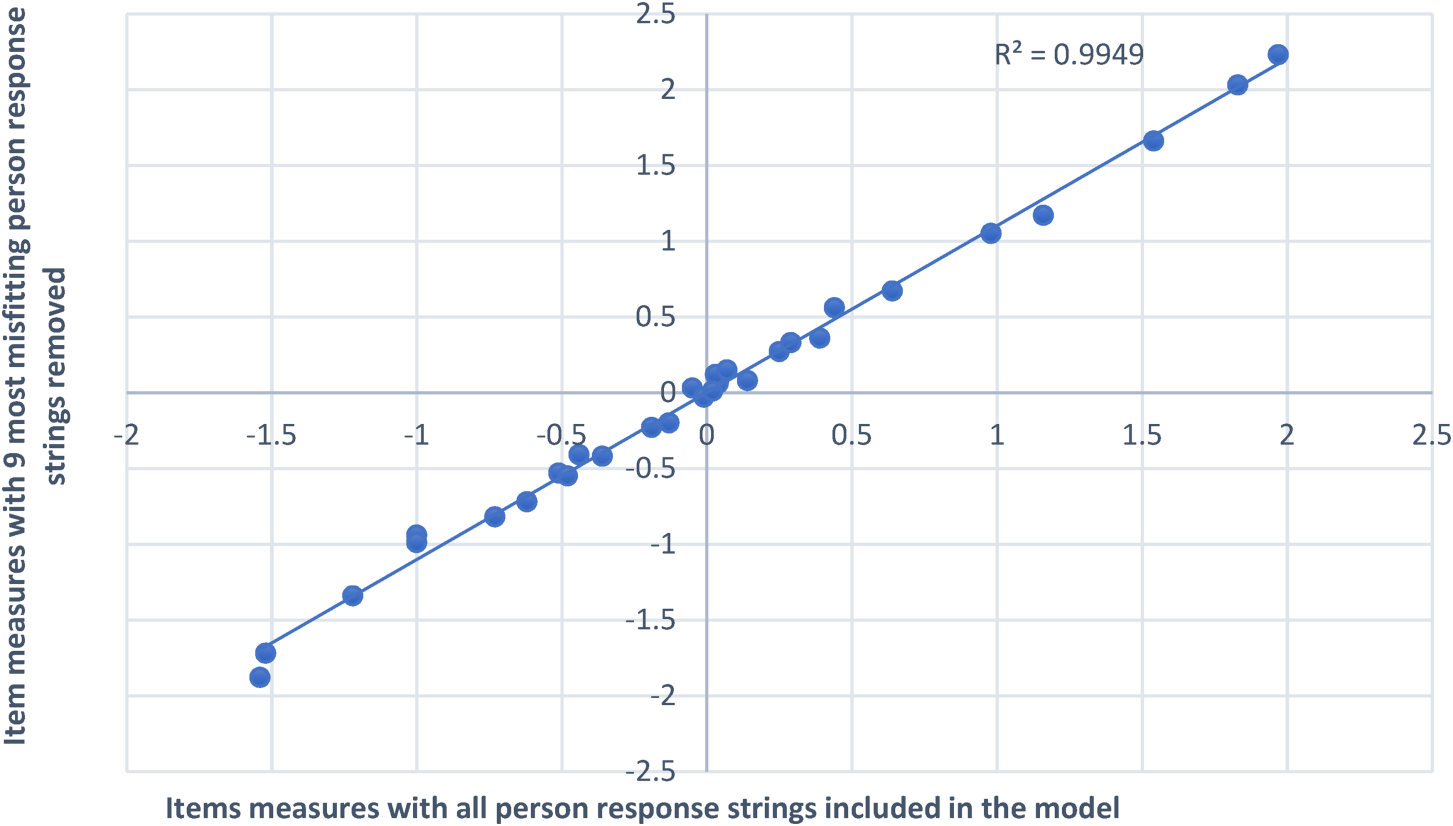
Comparing item difficulty measures with vs. without the 9 most misfitting person response strings in the model. The *x*-axis of this figure displays item difficulty measure estimates with all person response strings included in the model. The *y*-axis displays item difficulty measure estimates when the nine most misfitting person response strings were removed from the model. Item difficulty measures were very highly correlated (R = 0.997) for both scenarios (with vs. without the most misfitting person response strings). The line of best fit (the upward slanting dotted line) for both datasets is very close to the original item measure estimates (with all persons included in the model). Therefore, all person response strings were left in the final analysis model.

Person fit scores from 26 of the 177 assessments (15%) in this sample misfit the Rasch model. Assessment scores for the nine (5%) most misfitting person response strings (i.e., tests for a single day) were removed from the model and item measures and item difficulty measures were compared with and without these persons. Figure 2 shows that item difficulty measure estimates with and without the nine most misfitting person response strings in the model were very highly correlated (R = 0.997). Therefore, misfitting persons did not affect item difficulty measures or the item hierarchy.

#### Item difficulty and person-item match

**Figure 3:**
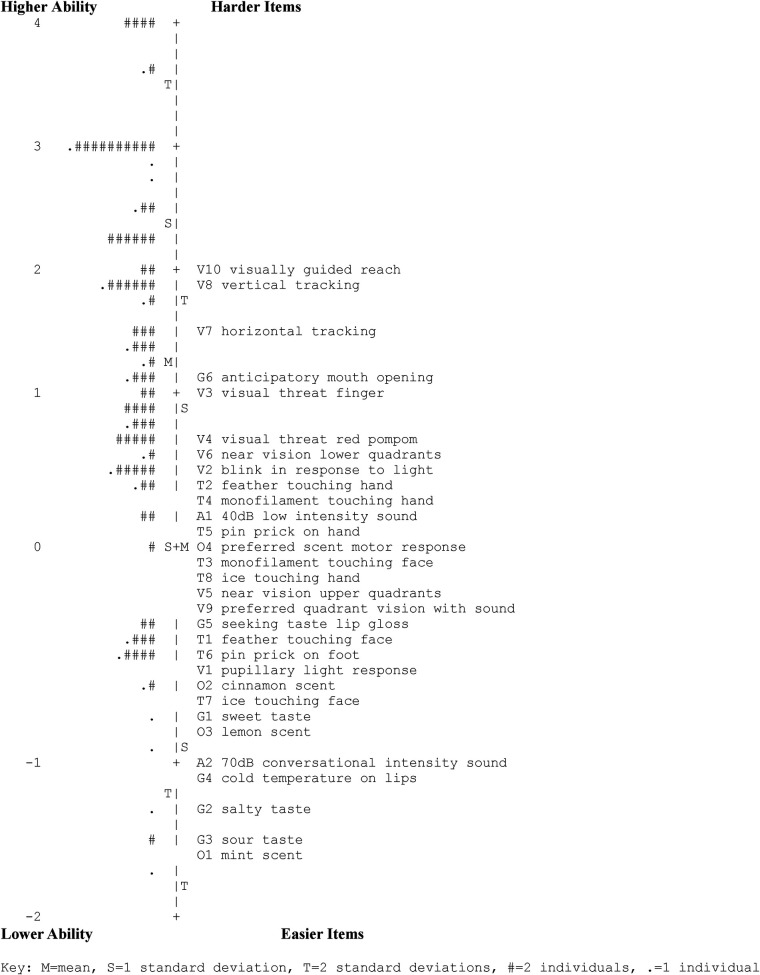
PASMA person-item Map. Numbers on the left side of the map (ranging from −2 to 4) are logits that represent measurement of person ability (left of the vertical dotted line) and item difficulty (right of the vertical dotted line). The lowest person ability and easiest items are shown at the bottom, and the highest person ability and hardest items are shown at the top. The Rating Scale Model anchors the mean item difficulty estimate for a sample to a logit value of 0. Therefore, items with a logit of 0 (i.e., O4 preferred scent motor response) represent the average item difficulty. This map shows that the easiest PASMA item is O1 mint scent and the hardest item is V10 visually guided reach. Left of the vertical dotted line, the lowest symbol (.) represents the participant with the least ability; the highest symbol (####) represents the participants with the most ability. Within this sample, the mean person ability was 1.35 logits higher than the mean PASMA item difficulty (anchored at 0 logits).

The person-item map (Figure 3) displays the distribution of all persons and items on the same linear scale. The range of distribution of person ability and item difficulty in our sample was six logits (−2–4). Average item difficulty is anchored at 0 logits by the Rating Scale Model, and average person ability level in our sample was 1.35 logits (SE = 0.46), which was >1 standard deviation higher than the average item difficulty. The easiest item on the PASMA was O1 mint scent (−1.54 logits, model SE = 0.25), and the most difficult item was V10 visually guided reach (1.97 logits, SE = 0.12).

The PASMA had high person reliability (0.81) and high item reliability (0.97). Zero of 177 assessment score totals (0%) received the minimum possible score, and eight of 177 (4.5%) received the maximum possible score on the PASMA. Thus, floor and ceiling effects were not observed.

### Differential item functioning

**Figure 4:**
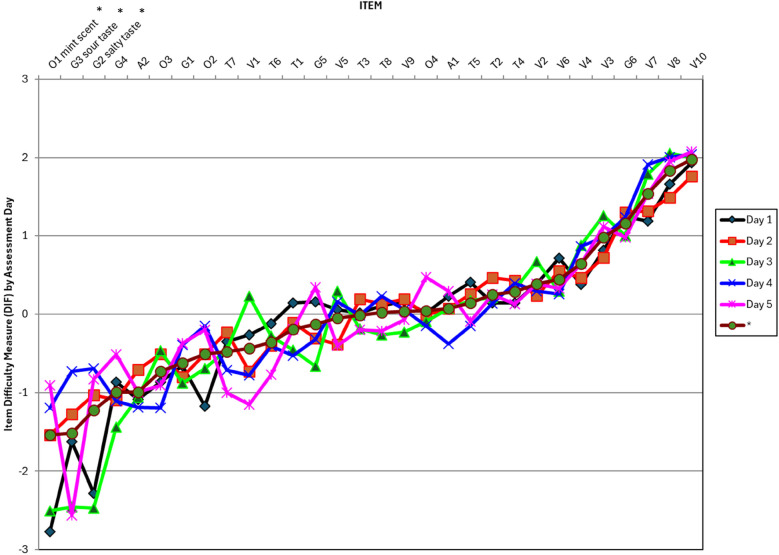
Differential item functioning across PASMA assessment days. PASMA items are listed in difficulty order from the easiest (on the left) to the hardest (on the right). Item difficulty estimates for each assessment day (1 through 5) are shown on the plot in the color/shape that corresponds with the key on the right side of the figure. The maroon line with a circle on the plot (shown below Day 5 on the figure legend) represents the item difficulty measure estimate from all assessment days combined. *Full items names are shown for the three items with differential item functioning across assessment days (O1 mint scent, G3 sour taste, and G2 salty taste). These items are the easiest on the PASMA (based on measure estimates for all assessment days combined) and their item difficulty was significantly lower on the days with differential item functioning (represented by the lowest points on the *y*-axis between −2 and −3).

Twenty-seven PASMA items (90%) functioned consistently when examined across five assessment days, but three items (i.e., O1 mint scent, G2 salty taste, and G3 sour taste) showed differential item function on specific days, as shown in Figure 4. These are the three easiest items on the assessment, meaning that participants were most likely to notice/respond to these olfactory and gustatory sensory stimuli. On some test days, participants responded less than expected to these three items, but even then, the items were among the easiest on the PASMA.

### Separation index

The person separation index was 2.06, indicating that the PASMA differentiated individuals in our sample into 3.08 statistically distinct strata.

## Discussion

Findings from this study demonstrate that the PASMA is a unidimensional measure that can be used to quantify sensory and motor awareness in children with medical complexity.

### Dimensionality

A secondary dimension consisting of visual items of the PASMA was identified, and these items could be explored as an independent measurement scale in future study. However, the Rasch analysis findings show that keeping visual items in the PASMA and treating all sensory sections as a single construct does not degrade the assessment's measurement characteristics. Clinically, keeping all PASMA items together enables the examiner to have one unified measurement value for a child's overall sensory and motor functioning which can guide holistic participation-based interventions. Therefore, we do not recommend removing any items at this time. This decision is supported by Andrich's metaphor of measuring student achievement in mathematics: Achievement can be subdivided into areas such as addition, subtraction, multiplication, and division and tested separately; or they can be grouped together on a single test to provide a broader measurement of mathematical achievement (30).

### Item difficulty hierarchy

The item hierarchy (shown on the right side of the person-item map in Figure 3) shows that the easiest PASMA items examine participants' responses to intense discreet sensory inputs (e.g., demonstrating awareness of smells such as mint, lemon, and cinnamon; concentrated sour, salty, and sweet flavors; oral tactile sensations such as cold temperatures; hearing their name spoken by a familiar person; and pupils reflexively constricting in response to light shining into eyes).

More challenging items included more subtle sensory inputs (e.g., whispering the child's name or touching the child's hand or face with a feather or pipe cleaner). Consistent hierarchies emerged within sensory sections. For example, demonstrating awareness of touch sensations on the face was relatively easy (i.e., a feather, a pipe cleaner, and ice), but demonstrating awareness of touch sensations on the hands was found to be more challenging. Patterns of tactile awareness were also identified: Noticing a pin prick stimulus on the hand was the easiest, whereas noticing a pipe cleaner or a feather touching the hand was more difficult.

The most difficult PASMA items were those that required participants to demonstrate a higher level motor response to sensory stimuli (e.g., moving away from approaching visual threats; opening mouth in response to a preferred flavor being offered; purposeful visual pursuits such as horizontal and vertical tracking to follow an engaging toy; and purposeful reaching toward the toy).

Overall, the item hierarchy derived from this analysis is consistent with clinical and theoretical expectations of sensory awareness and motor responses. Collectively, findings from this analysis suggest that this measurement scale is a conceptually valid representation of the construct of sensory and motor awareness among children with medical complexity.

### Item fit statistics

As shown in Table 6, Rasch analysis of the PASMA revealed that one item (V2 blink in response to light) misfit criteria for outfit. However, since this misfitting item does not degrade the measurement properties of the PASMA, we recommend keeping all items in the assessment to preserve fidelity to the original construct validity of this assessment. Item V2 takes less than ten seconds to administer, and it provides important information about whether the blink reflex is intact. This information can inform safe and comfortable participation in activities where noxious visual stimuli may be present. For instance, for children who do not reflexively blink in response to light, protective measures (e.g., protective eyewear, reduced bright lights/glare, and/or lubricating eye drops) could promote enjoyable participation in meaningful activities in their home, school, and community settings.

### Person-item match

The average person ability in this sample (1.35 logits) was higher than the average item difficulty (anchored at 0 logits by the Rasch model), but person-item match was adequate. The range of difficulty of PASMA items was sufficient to cover the spread of person ability in this sample, which was a heterogeneous group of children with medical complexity that was representative of the range of abilities observed among this population in clinical practice. Therefore, the item difficulty spread is believed to be sufficient to measure the wide range of sensory and motor awareness in the intended population of children.

### Separation index

The person separation index showed that the PASMA divided this sample into 3.08 statistically distinct person strata, meaning participants were separated into three groups based on sensory awareness and motor responsiveness: a group with low responsiveness, medium responsiveness, and the highest responsiveness (17). This distribution will be useful for identifying and understanding subgroups of sensory and motor awareness and may help to quantify changes (increases or decreases) in awareness which may occur in response to health status, medication, or rehabilitation.

Further exploration is needed to understand the cause of differential item function and determine whether this finding supports using fewer than five PASMA administrations per individual. For instance, if participants responded less to these items on later assessment days due to learning/habituation to the sensory stimuli, then fewer test administrations could reduce habituation, and thereby, reduce differential item functioning across days. Fewer PASMA administrations per individual could make using the assessment in clinical practice more feasible by decreasing the burden of testing on patients and therapists. However, because children with medical complexity commonly have variable alertness and responsiveness day to day, more research will be needed to demonstrate that the final recommended number of administrations (e.g., three) is sufficient to understand individuals' sensory and motor awareness and unique sensory and side preferences.

### Study limitations

First, this study is a secondary analysis of data that was collected for purposes other than examining item-level measurement properties. As such, some dataset factors were not optimal for this analysis. For example, in the analysis we used only the best score for items that are tested on both sides of the body. This represents the individual's sensory and motor awareness in the most ideal conditions (i.e., when stimuli are presented on their preferred side). We believe this scoring system provides maximum opportunity for therapists and caregivers to recognize each individual's rehabilitative potential. However, for children with medical complexity, sensory and motor awareness may be lower when conditions are not ideal (e.g., when stimuli are presented on the non-preferred side). Although this discrepancy is represented qualitatively on the PASMA score sheet (i.e., by showing point values for each side and noting observed side preference), it is not represented in the PASMA scores used in this Rating Scale Analysis.

Second, while the heterogeneous sample promotes generalizability of findings to a wide range of children with medical complexity between five and 21 years old, our findings are not generalizable to individuals with medical complexity outside of this age range. Additionally, factors that caused individuals to misfit the model expectations are not well understood, but our findings may be less generalizable to this misfitting subset of children with medical complexity.

### Potential impact of the instrument

The PASMA enables detection of subtle motor responses to sensory input. This information will help therapists and caregivers to set appropriate goals, design individualized interventions, and detect changes in the child's awareness and responses over time.

Perhaps most importantly, the PASMA provides information about a child's unique sensory strengths and preferences. This information can readily be applied to choose activities that suit the individual, to promote meaningful and enjoyable participation in daily activities. For example, a child who responds positively to olfactory and gustatory stimuli may enjoy being involved in cooking activities. A child who responds positively to tactile stimuli may enjoy water-based activities or hippotherapy.

Further, information from the PASMA can be applied to set up activities and environments to promote optimal participation. For instance, for a child participating in hippotherapy, knowledge of whether they respond more to stimuli on one side of the body will inform the therapist's position and how the horse's movements are directed. Auditory and visual environments of the activity can also be modified to meet the child's unique needs to make participation as immersive and enjoyable as possible (e.g., using an ideal voice volume and the best color for the horse's reigns and saddle). Importantly, knowledge of a child's sensory preferences can also be applied to everyday environments to help the child feel more regulated, comfortable, and safe.

### Future research

This study has established conceptual and structural validity of the PASMA using methodology informed by the RULER statement (33). Additionally, a degree of item measure reliability was established by examining differential item functioning across the five days that the PASMA was administered, but more research is needed to assess the reproducibility of these results and to address items that have differential function across assessment days. Future research is also needed to establish rater reliability and external validity of the PASMA. Future research will also examine consequential validity and will be geared toward optimizing use of PASMA results to guide rehabilitation decision-making for children with medical complexity.

## Conclusions

Although opportunities for future study have been described and will further enhance measurement properties of the PASMA, overall, this Rasch analysis provides support for this assessment's item-level measurement properties. The PASMA is a unified measurement tool which captures subtle signs of awareness of and response to sensory stimulation across domains in children with medical complexity. Future work will examine reliability and validity of the PASMA and will examine how baseline knowledge from this instrument can be used to guide individualized interventions, promote meaningful and enjoyable participation, and monitor changes over time for children with medical complexity.

## Data Availability

The raw data supporting the conclusions of this article will be made available by the authors, without undue reservation.
